# Barriers to the Use of Mobile Health in Improving Health Outcomes in Developing Countries: Systematic Review

**DOI:** 10.2196/13263

**Published:** 2019-10-09

**Authors:** Clemens Kruse, Jose Betancourt, Stephanie Ortiz, Susana Melissa Valdes Luna, Inderdeep Kaur Bamrah, Narce Segovia

**Affiliations:** 1 School of Health Administration Texas State University San Marcos, TX United States

**Keywords:** health outcomes, telemedicine, text messaging, communication barriers, developing countries, treatment outcome

## Abstract

**Background:**

The use of mobile health (mHealth) technologies to improve population-level health outcomes around the world has surged in the last decade. Research supports the use of mHealth apps to improve health outcomes such as maternal and infant mortality, treatment adherence, immunization rates, and prevention of communicable diseases. However, developing countries face significant barriers to successfully implement, sustain, and expand mHealth initiatives to improve the health of vulnerable populations.

**Objective:**

We aimed to identify and synthesize barriers to the use of mHealth technologies such as text messaging (short message service [SMS]), calls, and apps to change and, where possible, improve the health behaviors and health outcomes of populations in developing countries.

**Methods:**

We followed the Preferred Reporting Items for Systematic Reviews and Meta-Analyses checklist. Deriving search criteria from the review’s primary objective, we searched PubMed and CINAHL using an exhaustive terms search (eg, mHealth, text messaging, and developing countries, with their respective Medical Subject Headings) limited by publication date, English language, and full text. At least two authors thoroughly reviewed each article’s abstract to verify the articles were germane to our objective. We then applied filters and conducted consensus meetings to confirm that the articles met the study criteria.

**Results:**

Review of 2224 studies resulted in a final group of 30 articles for analysis. mHealth initiatives were used extensively worldwide for applications such as maternal health, prenatal care, infant care, HIV/AIDS prevention, treatment adherence, cardiovascular disease, diabetes, and health education. Studies were conducted in several developing countries in Africa, Asia, and Latin America. From each article, we recorded the specific health outcome that was improved, mHealth technology used, and barriers to the successful implementation of the intervention in a developing country. The most prominent health outcomes improved with mHealth were infectious diseases and maternal health, accounting for a combined 20/30 (67%) of the total studies in the analysis. The most frequent mHealth technology used was SMS, accounting for 18/30 (60%) of the studies. We identified 73 individual barriers and grouped them into 14 main categories. The top 3 barrier categories were infrastructure, lack of equipment, and technology gap, which together accounted for 28 individual barriers.

**Conclusions:**

This systematic review shed light on the most prominent health outcomes that can be improved using mHealth technology interventions in developing countries. The barriers identified will provide leaders of future intervention projects a solid foundation for their design, thus increasing the chances for long-term success. We suggest that, to overcome the top three barriers, project leaders who wish to implement mHealth interventions must establish partnerships with local governments and nongovernmental organizations to secure funding, leadership, and the required infrastructure.

## Introduction

### Background

Mobile devices are a cheap source of technology for addressing health care needs in developing countries. With the expansion of technology, mobile health (mHealth) is a tool that can be used to exchange health information for improving health outcomes through short message service (SMS) text messaging, mobile apps, and calls [1]. mHealth offers simplicity, efficiency, and effectiveness to patients due to its ability of rapid communication. mHealth intervention is a useful tool due to the ability to be accessible at the user’s convenience. Mobile apps can be used to assess and measure the impact of a specific disease or may actually prevent a specific illness from occurring. A simple text can communicate, store, retrieve, and remind patients of their health status or deliver messages that promote healthy behaviors and choices. It is an inexpensive tool that can reduce the disparities of health in developing countries. Health care professionals now use smartphones or tablet computers to accomplish tasks for which they once used to need a pager or a personal digital assistant [2,3].

### Definition of Key Terms

The term barrier is defined as “something that separates one thing from another” [4]. It is anything that prevents a certain goal from being achieved [4]. The second term, mHealth, is defined as a “medical and public health practice supported by mobile devices, such as mobile phones, patient monitoring devices, personal digital assistants, and other wireless devices” [5]. A developing country is a country that has a slow rate of industrialization, low per capita income, high unemployment, high poverty rate, and low standard of living [6]. Developing countries usually rely on developed countries for their economic growth and prosperity [6].

### Rationale for the Review

mHealth is a tool that has had a positive impact on developed countries and has contributed to improving the health outcomes of populations around the world [7]. Specifically, researchers have focused on SMS in health care, and leading health organizations recommend its use [8]. Around the globe, mobile-cellular subscriptions will soon match the number of the population worldwide and are expected to continue to increase [9]. This is especially true in the developing world, where the market has not yet reached saturation [9]. mHealth has closed the gap in the digital divide in low-resource areas [10]. In the developing world, the World Health Organization reported a shortage of health care workers in 57 countries, resulting in a clear opportunity for innovative and effective solutions to help improve the health outcomes of their most vulnerable populations [11]. Mobile technology devices such as tablets, phones, computers, and tracking devices can be used to support and enhance health care in developing countries. The use of text messaging to promote healthy behaviors and healthy choices can be considered a groundbreaking component in improving population and community health.

### Context of Other Evidence

Many studies have been carried out to determine the efficiency of mHealth in developing countries. A literature review of SMS-supported interventions for surveillance, management, treatment compliance, and prevention of noncommunicable diseases in India, South Africa, and Kenya found mobile phones to be well accepted by the population; however, high-quality intervention designed studies were needed [12]. In Nigeria, mobile device questionnaires were used to understand the perceptions of women at high risk of maternal death; although over 90% of women owned mobile phones, innovative methods were lacking to strengthen the delivery of maternal health information to those hard-to-reach populations [13]. In Zambia, SMS was found to have the potential to diagnose HIV early in infants by accelerating the delivery of results of blood sample testing to clinics, but the identification of lack of mobile phone ownership during the design of the study was found to hinder the success of the intervention [14].

In China, a smartphone app and text messaging were used to improve vaccination coverage among children, as well as the consumption of infant micronutrient powder packets [15]. Caregivers’ suspicious beliefs and lack of acceptability were the major causes negatively affecting the success of the mHealth intervention [16,17]. In remote areas of Vietnam, the mMom app was used to improve pregnant women’s maternal and infant health knowledge. An anticipated challenge was the high level of integration among local partners that required constant communication and engagement for coordination of the mHealth initiative [3].

An exploratory qualitative study conducted in Latin America and the Caribbean sought to further understand the needs of underserved populations and their exposure to public health interventions that used information and communications technologies to highlight the scarcity of such tools to reduce inequities. The greatest challenges were the lack of sustainability for financial and technical resources due to the unreliability of sustained external funding, poor intervention design caused by the resistance of precedents, and lack of technological literacy among participants unfamiliar with the use of information and communications technologies [18].

A study designed for primary prevention of hypertension in Argentina, Guatemala, and Peru found challenges that consisted of the unacceptability of mHealth innovations by the targeted communities, and emphasized the need to tailor the interventions to potential literacy challenges attributed to lack of understanding of cultural context [19]. In Brazil, a mobile phone–based intervention to promote prenatal care practices found that only one-fifth of women eligible for the study were actually interested in participating [19]. In Tajikistan, Bolivia, and Palestine, a behavioral change intervention was deployed using text messages to bring awareness of using contraceptives among the young to prevent unwanted pregnancies [20]. Negative attitudes toward and beliefs about contraception, including the cultural stigma of having sex before marriage, being judged, and confidentiality concerns, limited participant discussion of contraception with providers [21]. In urban and rural areas of Guatemala, text messages were used to remind parents of infants to attend vaccination visits and decrease unnecessary morbidity [22]. This study concluded that client preference for delivery modalities such as a combination of text messaging and phone calls should have been considered to reach the maximum amount of the targeted population [22].

### Objective

While other studies have focused on the potential benefits of mHealth in developing countries, our literature review sought to define the barriers that impede the successful application of mHealth (eg, SMS, cell phones, apps) interventions that aim to improve health outcomes of a population in diverse developing countries around the world. This knowledge may serve as a useful tool for project leaders to consider when planning or designing future mHealth interventions to strengthen the chances of long-term, sustainable success in the community. To analyze the use of mHealth in developing countries, more studies need to examine the different types of barriers these countries face. We conducted this literature review to determine what type of mHealth initiatives are more popular in developing countries, as well as the outcomes and barriers identified by the respective article authors. We aimed to provide a clearer understanding of what initiatives have the best supporting evidence of improving health outcomes by using mHealth approaches and of the resources developing countries require to foster the long-term and sustainable success of these projects.

## Methods

### Protocol Registration and Eligibility Criteria

This review followed the Preferred Reporting Items for Systematic Reviews and Meta-Analyses (PRISMA) guidelines (Multimedia Appendix 1) [23]. We did not register the review. The main objective was to identify and synthesize the barriers to the use of mHealth to improve the health outcomes in developing countries. Articles were eligible for review if they met criteria such as having a health outcome and involving the use of mHealth technology in a developing country. We analyzed articles only if (1) the full-text article was available, (2) the article related to humans, (3) the article was published between 2008 and 2018, and (4) the article was written in English. Exclusion criteria for this review were systematic reviews, articles unrelated to the objective of the review, and no direct health outcome being involved. We did not consider for this review any studies that were in progress. Finally, we removed duplicate articles from the literature matrix.

### Information Sources and Search

In addition to reporting this review in accordance with the PRISMA guidelines, we conducted it using techniques of the Assessment of Multiple Systematic Reviews [24]. We searched PubMed and CINAHL with an exhaustive search string comprising Medical Subject Headings (MeSH), due to their widespread availability, and Boolean operators. Multimedia Appendix 2 provides a detailed list of MeSH terms. We conducted searches for this literature review in September 2018.

### Risk of Bias in Individual Studies

Our methods did not enable randomization, so to control for selection bias, we conducted the search using exhaustive MeSH terms, which are widely used in databases used for literature research. To avoid influencing each other’s individual opinions, each researcher recorded his or her findings independently. We calculated a kappa statistic to measure interrater reliability, which refers to the agreement and consistency of article selection. The kappa was .78, which reflects a moderate agreement between the reviewers [25,26].

## Results

### Study Selection, Data Collection Process, and Data Items

**Figure 1:**
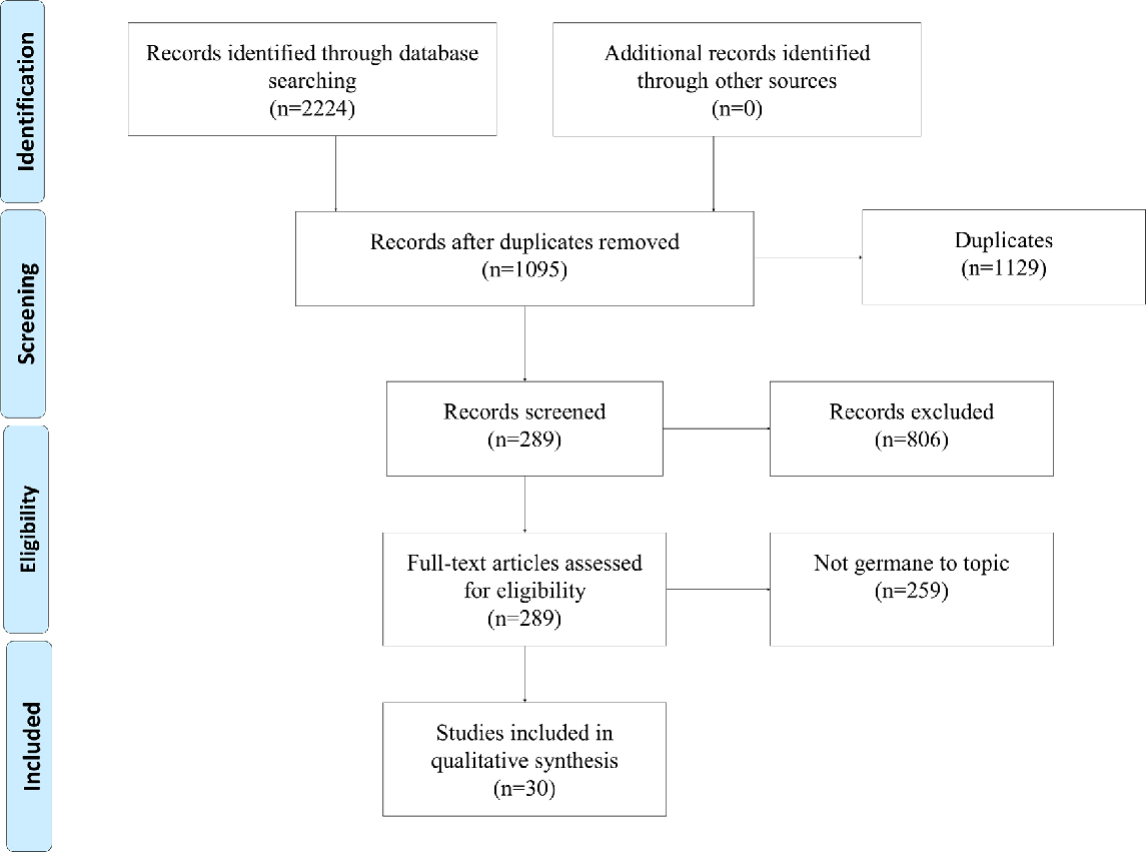
Preferred Reporting Items for Systematic Reviews and Meta-Analyses flow diagram of the literature search and selection process.

Figure 1 illustrates the search and selection process in each database. The initial search in PubMed resulted in 535 articles, and in CINAHL the search produced 1689 articles. The total number of articles obtained from both search engines was 2224. Then, after applying limiters, we narrowed the PubMed results down to 144 articles and CINAHL to 145 articles. All 289 abstracts were screened by at least two reviewers. Reviewers screened abstracts recording their individual observations and recommendations to either include or exclude each article. A consensus meeting was then held to arrive at an agreement on the selected articles. If there was a disagreement, a third author’s vote was required to reach consensus. We produced a list of 58 abstracts germane to the objective of the review. We distributed these articles among our team in a manner that ensured each article was analyzed by at least two reviewers. A second consensus meeting arrived at a final group of articles for analysis of 30. The other articles were eliminated after a full reading.

### Study Selection and Characteristics

Between September and November 2018, we reviewed the 30 articles germane to this review’s objective of defining the barriers to implementing mHealth interventions in developing countries. Results included mHealth initiatives covering health outcomes such as prenatal care, infectious diseases, medication adherence, appointment reminders, and chronic diseases education. The articles in the group for analysis reported interventions piloted across several developing countries around the world. We divided the 30 articles among the reviewers for analysis by at least two reviewers. We held group consensus meetings to facilitate discussion of individual barriers identified in the articles.

### Results of Individual Studies and Synthesis of Results

We analyzed the articles to determine the health outcomes most commonly improved with mHealth interventions, the most used mHealth technology, and, most importantly, the barriers that hindered the adoption or impact of the mHealth interventions. We break down each of the findings to provide a comprehensive vision for future leaders who wish to implement mHealth interventions in developing countries.

Among the articles analyzed, maternal health was the most prevalent health outcome with a frequency of 9 out of the 30 articles (30%) [27-35]. Infectious diseases and chronic diseases were the second most prevalent health outcomes, with a frequency of each of 8 of 30 (27%) articles [36,39-44,49-51]. The third most prevalent outcome was preventive health, occurring in 5 of 30 (17%) articles [22,52-55].

The mHealth interventions identified in the literature were SMS only; SMS or phone calls and voice messages, or both SMS and phone call and voice messages; SMS phone app; multimedia messages for diagnosis; and a combination of SMS, smartphone app, and cellphones. We identified SMS in 28 of the 30 articles we analyzed (93%). The use of SMS only was mentioned in 16 of 26 interventions (62%) [28-31,35,37,38,40-43,45-47,50,52]. SMS with or without phone calls and voice messages was the second highest with a frequency of 4 of 26 interventions (15%) [22,32,39,53]. Smartphone with the use of apps was the third most commonly mHealth used with a frequency of 3 of 26 interventions (12%) [34,36,55]. Interventions using multimedia messages had a frequency of 2 of 26 (4%) [27,54]. Finally, the combination of SMS, smartphone app, and cell calls occurred in only 1 of 26 (4%) interventions [33].

We classified the 73 barriers into 14 categories identified in the 30 articles. The categories are immature (or lack of) infrastructure (10/73, 14%), lack of equipment (9/73, 12%), technology gap (9/73, 12%), human resource issues (7/73, 10%), time or work conflict (7/73, 10%), cost (6/73, 8%), lack of public policy (8/73, 8%), literacy (4/73, 5%), language barriers (4/73, 5%), psychosocial issues (4/73, 5%) lack of training (3/73, 4%), concerns about privacy and confidentiality of information (2/73, 3%), lack of efficacy (1/73, 1%), and exposure of program (1/73, 1%).

**Figure 2:**
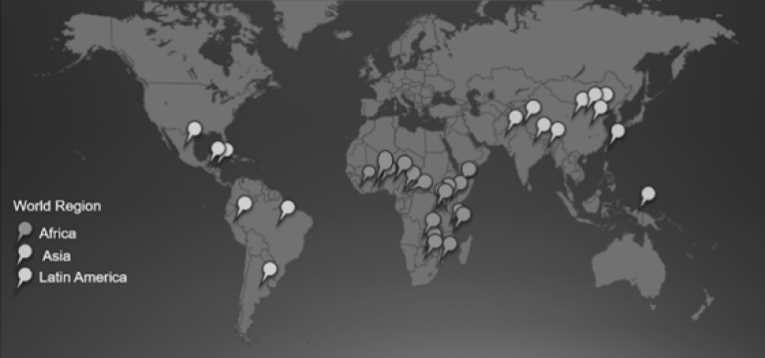
Location of studies of mobile health technologies to improve preventive health outcomes in developing countries, by region.

Figure 2 provides a geographic distribution of the studies in the analyzed studies. Africa accounted for 16 of 30 (53%) articles, Asia for 10 of 30 (33%) articles, and South America for 3 of 30 (10%) articles; 1 article studied both Africa and South America (3%).

Table 1 summarizes the following details of each article analyzed: study design, sample size, technological intervention, health outcome, barriers identified, and world region.

**Table 1:** Summary of results.

First author, date, reference	Study design and sample size	mHealth intervention category	Health outcome category	Barriers identified	Continent
Ginsburg, 2015 [36]	Open data-kit survey (design-stage evaluation activity)	Smartphone app	Infectious disease	Technology gap Language barrier	Africa
Nhavoto, 2017 [37]	RCT^a^ and interviews (tuberculosis n=69, HIV n=72)	SMS^b^ only	Infectious disease	Privacy concerns Literacy Language barrier Equipment	Africa
Bediang, 2014 [38]	Blinded RCT (intervention n=104, control n=104)	SMS only	Infectious disease	Infrastructure Cost Policy	Africa
Bigna, 2013 [39]	RCT (n=224 divided into 4 groups)	SMS with or without phone calls and voice mail	Infectious disease	Equipment Language barrier Policy Privacy concerns Infrastructure	Africa
Medhanyie, 2015 [27]	Interviews, 2893 electronic health records of 1122 women	Multimedia messages for diagnosis	Maternal health	Time or work conflict Human resources issues Time or work conflict Time or work conflict	Africa
Rokicki, 2017 [28]	Cluster RCT (n=756)	SMS only	Maternal health	Time or work conflict Equipment Cost	Africa
Toda, 2016 [52]	Clustered RCT (intervention n=32 [88 cases], control n=32 [21 cases])	SMS only	Preventive health	Human resources issues Technology gap Training	Africa
Flax, 2017 [29]	Cluster RCT and interviews (n=195)	SMS only	Maternal health	Infrastructure Psychosocial stressors	Africa
Ngabo, 2012 [30]	Pilot study	SMS only	Maternal health	Infrastructure Equipment Cost	Africa
Jia, 2015 [53]	Longitudinal data analysis of monthly electronic health record suspect and mortality cases for both the traditional sentinel program and mobile phone reporting (n=178)	SMS with or without phone calls and voice mail	Preventive health	Training	Africa
Leon, 2015 [40]	RCT (n=22 studied, n=15 interviewed)	SMS only	Chronic disease	Technology gap Infrastructure Psychosocial stressors	Africa
Hao, 2015 [41]	Interviews (n=11)	SMS only	Infectious disease	Time or work conflict Equipment Policy	Africa
Lund, 2014 [31]	Open-label, pragmatic-cluster RCT, 2550 pregnant women (intervention n=1311, control n=1239)	SMS only	Maternal health	Equipment Literacy	Africa
Tuijn, 2011 [54]	Feasibility study attaching a cell phone to a microscope	Multimedia messages for diagnosis	Preventive health	Infrastructure Equipment Training	Africa
Linnemayr, 2017 [42]	True experiment (n=332)	SMS only	Infectious disease	Technology gap	Africa
Steury, 2016 [43]	RCT (n=96)	SMS only	Chronic disease	Efficacy Cost	Africa
Ippoliti, 20107 [32]	RCT (n=17)	SMS with or without phone calls and voice mail	Maternal health	Infrastructure Literacy	Africa and South America
Uddin, 2017 [33]	Stratified 2-stage random-cluster sampling technique to select participants (n=5280)	SMS, smartphone app, and cell phone calls	Maternal health	Technology gap	Asia
Balakrishnan, 2016 [34]	Feasibility study (n=512)	Smartphone app	Maternal health	Cost Training	Asia
Fang, 2018 [44]	RCT (n=247)	SMS with or without phone calls and voice mail	Chronic disease	Technology gap Equipment Psychosocial stressors	Asia
Zhou, 2016 [45]	RCT and the intervention period lasted for 6 months and used baseline and follow-up surveys (n=351 villages, n=1393 children)	SMS only	Chronic disease	Policy	Asia
Fang, 2016 [46]	Random sampling method RCT (3 groups: SMS [n=95] SMS + Micro Letter app [n=92], and phone [n=93])	SMS only	Chronic disease	Technology gap Infrastructure	Asia
Kumboyono, 2017 [47]	No RCT, posttest-only control group design (n=90)	SMS only	Infectious disease	Psychosocial stressors	Asia
Kazi, 2013 [48]	No RCT, systematic random sampling (n=28)	SMS only	Infectious disease	Human resources issues	Asia
Mohan, 2017 [49]	Cross-sectional study (n=800)	SMS only	Chronic disease	Language barrier	Asia
Lin, 2017 [35]	True experiment (n=757)	SMS only	Maternal health	Training Policy Exposure of program Time or work conflict	Asia
Wu, 2014 [55]	Prospective true experiment (intervention n=97, control n=128)	Smartphone app	Preventive health	N/A^c^	Asia
Beratarrechea, 2015 [22]	Interviews (n=43)	SMS with or without phone calls and voice mail	Preventive health	Infrastructure Technology gap	South America
Rico, 2017 [50]	Interviews (n=14)	SMS only	Chronic disease	Literacy Technology gap	South America
Piette, 2012 [51]	RCT (n=200)	SMS only	Chronic disease	Cost Equipment Infrastructure Policy	South America

^a^RCT: randomized controlled trial.

^b^SMS: short message service.

^c^N/A: not available.

## Discussion

### Principal Findings

This review identified the common barriers faced by developing countries in the adoption of mHealth. mHealth is widely used in developing countries as a tool to improve the health outcomes of highly vulnerable communities and individuals. Based on the evidence found in this review, mHealth is an effective method to support health care services. mHealth has been used in many developing countries in regions such as Africa, Asia, and Latin America. These countries constantly battle infectious diseases, chronic diseases, perinatal complications, acute diseases, birth defects, and many more. This review revealed important barriers that must be understood before implementing mHealth initiatives. Considering and assessing these barriers prior to the design phase of an mHealth intervention will have a positive impact on the health outcomes of populations and individuals.

As noted above, mHealth can provide a great opportunity to solve health care issues faced by developing countries. However, there are various challenges and barriers to be considered prior to implementation.

#### Health Outcomes

Based on the findings of this review, the 2 main health outcomes affected by an mHealth intervention were infectious diseases and maternal health. In developing countries, the burden of infectious diseases is prevalent due to poverty, leading to “poor nutrition, indoor air pollution and lack of access to proper sanitation, and lack of health education” [56]. According to the World Health Organization, most illnesses are avoidable and treatable. It is estimated that diseases account for up to 45% of the burden in poor countries due to poverty; HIV, tuberculosis, and malaria account for 18% [56]. The second most common health outcome affected by an mHealth intervention was maternal health. Developing countries account for 99% of all maternal deaths compared with developed countries [53]. This is the biggest health gap in the world [53]. In remote locations, poor women are more prone to receive inadequate care, specifically in the areas lacking skilled health care workers [27].

The majority of the reviewed articles used SMS as an mHealth intervention to improve infectious disease, health outcomes, and patient treatment adherence. In developing countries, infectious diseases are prevalent due to the lack of preventive care. mHealth interventions in developing countries were considered effective in improving antenatal care, vaccination, and preventive treatment for chronic and infectious diseases [55]. SMS was also effective for maternal health, prenatal care, infant care, HIV/AIDS prevention, treatment adherence, cardiovascular disease care, diabetes care, health education, tuberculosis prevention and care, anemia care, immunization, and disease awareness [32,49]. SMS directly increased disease awareness by providing health tips and reinforcing reminder systems. Moreover, SMS provided emotional support to patients, promoted knowledge about health, and influenced attitude change toward greater self-responsibility [36].

#### Mobile Health Tool Used

SMS was the most commonly used mHealth tool due to the number of mobile phones in use. An estimated 4.5 billion people are expected to have mobile phones worldwide by 2020 [33]. Compared with other methods of communication, text messaging has an advantage due to its low cost and high reliability [33]. Researchers in the field state that educational mHealth training programs are effective in raising awareness by offering an efficient and cost-effective way to achieve the success of mHealth implementation [42]. A health education approach via mobile phones can be used to manage diseases, aid medical testing, and improve treatments. Specifically, text messaging can be used for interventions and health education, because it is particularly popular in developing countries [42]. Through simple text messaging, patients have reported that they felt more confident in their treatment [49]. SMS has the potential to make patients feel supported, encouraged, and aware, thus helping them take better care of themselves and continue treatment [28]. SMS reminders also improve appointment attendance and SMS text messaging helps health care providers prescribe medicine on a timely basis, consequently improving patient care [32,39].

SMS is perceived as a tool that can boost the rate of adherence to medical treatment and has the potential to help in the prevention of diseases. Health information transmitted through text messages can also effectively be used to manage the treatment of infectious diseases such as tuberculosis [45]. The main mechanism is the use of text messages to remind patients about appointments and taking medications, to deliver motivational messages and health education or health promotion messages. Another approach is the use of mobile phones by health workers to help support services in diagnosing women and children in remote areas and identifying patients at risk who need to be referred. Throughout the literature review, SMS was often mentioned in combination with phone calls, voice messages, smartphone apps, and multimedia messages. However, the literature also provided examples of participants who did not know how to use mobile phones or similar technology efficiently. In other words, they did not know how to work a phone or were unable to read text messages. This limited knowledge may be attributed to the amount of exposure individuals in developing countries have to technology. Therefore, there was a correlation between frequency of phone usage and knowledge of this technology. Phone calls as an mHealth intervention can also help improve health outcomes while at the same time offering participants a simpler method than SMS. Mobile phone apps were proven to be an efficient tool to assist people in achieving early screening for diagnosis and treatment purposes. Moreover, apps have been shown to prevent health complications, thus helping improve preventive medicine [50].

#### World Region

Africa was the most frequent study setting in the articles we analyzed. This review suggested that this region has been extensively participating in mHealth projects.

The 3 most prevalent barrier categories were lack of infrastructure, lack of equipment, and technology gap. Developing countries should consider investing in their infrastructure and encouraging partnerships with equipment providers to help their populations afford phones and learn how to use them. It is vital for developing countries to adapt to new emerging technologies in an effort to reduce the risk of being left behind in the great technological advancements in health.

### Strengths and Limitations

This review had several limitations. First, selection bias tended to be prevalent in many research studies. To help address this problem, we held consensus meetings once per week to discuss the findings of the research articles. However, our main controls for selection bias were (1) identifying the research objective, (2) defining the key terms used, and (3) having more than one reviewer examine each article. We conducted all consensus meetings either through Skype or in person. These meetings offered great value to our research because they reduced personal bias when eliminating the articles from the literature matrix. We gathered feedback, opinions, and knowledge throughout the process. Another selection bias was the selection of only free full-text articles. We eliminated a few articles in this step. Including those articles in the review most likely would not have changed the outcome of our review, but it might have identified additional barriers.

Second, we examined only 10 years’ worth of articles when abstracting the data. However, this may or may not be a limitation, as the technology used in mHealth may not have existed earlier. A third possible limitation is publication bias for the 10 years considered in this review.

This review adds to the body of knowledge on the significant barriers mHealth confronts in developing countries. This review was constructed in accordance with PRISMA guidelines. We limited our review to 2 well-known research databases, CINAHL and PubMed. As a result, we expect this review will have a high external and internal validity.

### Recommendations and Suggestions for Further Research

To overcome these barriers, the published literature suggested some important solutions. Strengthening health care systems through the use of mHealth requires strong governance, as well as the commitment of the private sector [35,48]. More investment in phones and rigorous training on these devices is also required to improve their acceptability in developing countries [47]. It is also important to consider the characteristics of the population, such as socioeconomic background, to gain a better perspective of the community [29]. When phone ownership is lacking, a microcredit program, in which several people can obtain a loan to purchase a group phone, may be feasible, and consequently the group would rely on other family members or the community to improve their health [37]. The successful development of interventions using the capability of mHealth technologies lies within the criticality of mHealth research. It entails important characteristics, such as collaboration throughout all phases of the project [43].

It is important to adapt and redesign emerging interventions as the technology advances. The future of mHealth in both developed and developing countries is expected to be prosperous with new innovations arising exponentially throughout the health care domain. It is important to assess the disparities by country in order to improve their respective health care sectors. Community needs could be addressed and improved through the use of available technology by country. However, to drastically make a change and improve the use of mHealth in developing countries, policy reform at all levels is needed.

Project leader support through policy reform could compensate for the barriers faced in developing countries. Therefore, there is a need for future research on how governments can help their countries reach their goals to improve and increase the acceptance of mHealth as a means to improve health care and, ultimately, improve the health of their communities. In addition, there is a need for future implementation of mHealth technologies such as text messaging to improve chronic diseases, such as tuberculosis, HIV, hypertension, cardiovascular disease, colorectal cancer, and pneumonia, in remote and resource-limited settings to overcome the challenges a community faces. Implementation of mHealth initiatives requires rigorous training of health care workers, as well as of the designated population who will be participants in studies, to understand and use the technology correctly [39].

Training on the use of devices, such as cell phones and mobile apps, and on sharing and receiving text messages will not only improve the performance but also increase the acceptability of mHealth within the community [39,46]. Special attention needs to be paid to the illiterate when using SMS due to the inability of participants to read and comprehend these messages [55]. Lastly, there is a need to design the health system based on approaches to control the timing of text messaging, mobile network fluctuations, and mobile phone turnovers to improve treatment adherence and follow-up visits in cases of chronic diseases, infectious diseases, maternal care, and birth defects [32,48].

### Conclusion

The published literature demonstrates the barriers faced by developing countries in the use of mHealth to improve health outcomes. This systematic review shed light on the most prominent health outcomes that can be improved using mHealth technology interventions in developing countries. SMS technology is readily available at low cost in developing countries and can be easily adopted to interventions that improve the health outcomes already identified. Additionally, the barriers identified will provide the leaders of future intervention projects a solid foundation for the design of those interventions, thus increasing the chances of long-term success and sustainability. We suggest that, to overcome the top 3 barriers, project leaders who wish to implement mHealth interventions must establish partnerships with local governments and nongovernmental organizations to secure funding, leadership, and the required infrastructure. This research identified the barriers and the frequency of those barriers by region. It also identified the most used type of mHealth tool, as well as the health outcomes affected by the tool used. This literature review highlighted the need for policy reform in developing countries to improve health care and, ultimately, improve the health of their communities.

## Appendix

Multimedia Appendix 1 Preferred Reporting Items for Systematic Reviews and Meta-Analyses (PRISMA) checklist.

Multimedia Appendix 2 Key terms used in the search string.

## Abbreviations

MeSH: Medical Subject Headings
mHealth: mobile health
PRISMA: Preferred Reporting Items for Systematic Reviews and Meta-Analyses
SMS: short message service
