# Supplementary material for: Barriers to the Use of Mobile Health in Improving Health Outcomes in Developing Countries: Systematic Review
Source: J Med Internet Res. 2019 Oct 9;21(10):e13263. doi: 10.2196/13263 (PMC6811771; doi:10.2196/13263)
Supplement: Multimedia Appendix 2 [file jmir_v21i10e13263_app2.pdf]

Key terms used in search string.

("Messaging, Text" or "Texting" or "Textings" or "Short Message, Service" or "Text Messages" or "Message, Text" or "Messages, Text" or "Text Message") OR ("mHealth" or "Mobile Health" or "Health, Mobile" or "Telehealth" or "eHealth") OR ("Less-Developed Countries" or "Countries, Less-Developed" or "Country, Less-Developed" or "Less Developed Countries" or "Less-Developed Country" or "Under-Developed Nations" or "Nation, Under-Developed" or "Nations, Under-Developed" or "Under Developed Nations" or "Under- Developed Nation" or "Third-World Countries" or "Countries, Third- World" or "Country, Third-World" or "Third World Countries" or "Third- World Country" or "Third-World Nations" or "Nation, Third-World" or "Nations, Third-World" or "Third World Nations" or "Third-World Nation" or "Under-Developed Countries" or "Countries, Under-Developed" or "Country, Under-Developed" or "Under Developed Countries" or "Under-Developed Country" or "Developing Nations" or "Developing Nation" or "Nations, Developing" or "Less-Developed Nations" or "Less Developed Nations" or "Less-Developed Nation" or "Nation, Less-Developed" or "Nations, Less-Developed")
